# Computational psychiatry: a report from the 2017 NIMH workshop on opportunities and challenges

**DOI:** 10.1038/s41380-018-0063-z

**Published:** 2018-04-27

**Authors:** Michele Ferrante, A. David Redish, Maria A. Oquendo, Bruno B. Averbeck, Megan E. Kinnane, Joshua A. Gordon

**Affiliations:** 1 National Institute of Mental Health, National Institutes of Health, Bethesda, MD USA; 2 Department of Neuroscience, University of Minnesota, Minneapolis, MN USA; 3 Perelman School of Medicine, University of Pennsylvania, Philadelphia, PA USA

**Keywords:** Neuroscience, Psychology

Psychiatry is at a crossroads. Neuroscience has made substantial progress toward understanding the link between brain mechanisms and behavior, but incorporating this understanding into psychiatric practice has proven challenging. One potential way forward is to use computational techniques [1, 2]. Computational techniques can be harnessed to develop models that clearly link neural systems to behavior [3, 4]), allowing us to develop concrete theoretical conceptualizations that bidirectionally link levels of analysis, from molecules to circuits and from circuits to behavior [3, 5, 6]. In addition, to identify regularities and other consistent, identifiable features in large psychiatric data sets, computational techniques may enable identification, categorization, and prediction of dimensional processes in heterogeneous psychiatric populations [3, 7]. To determine how computational techniques and perspectives could inform psychiatric practice and how psychiatric studies could drive new directions in computational neuroscience, the National Institute of Mental Health (NIMH) organized a 2-day meeting (26–27 June 2017) to bring together leaders in experimental and computational neuroscience and clinical and translational psychiatry to discuss and define the opportunities and challenges that exist in the field of Computational Psychiatry (see the new Computational Psychiatry Program and the Theoretical and Computational Neuroscience Program at NIMH for related funding opportunities). Approximately 90 investigators and NIMH staff attended the workshop. The list of participants can be found here and detailed minutes of the meeting are available upon request.

The objectives of the workshop were threefold: to identify how Computational Psychiatry as a field can move forward; to foster the development of a community of scholars working in fields related to Computational Psychiatry; and to ensure the alignment of perspectives between researchers and NIMH staff to foster this nascent field.

Conversations were structured around targeted questions related to four main themes: identifying the overall goals of the field, looking at the field from the perspective of computation, looking at it from the perspective of psychiatry, and looking at it from the perspective of basic/fundamental science. As a summary of the program, we address each of these themes separately and then we integrate them into concrete suggestions to move the field forward.

## Goals of the field

The ultimate goal for psychiatric research is to provide clinical value by positively impacting quality of life, by improving the mental health of patients. Computational approaches could indeed impact clinical care, for example, by enabling better theory- and data-driven diagnoses as well as predictions of patient outcomes and specification of treatment options [7]. However, interim successes for researchers, clinicians, and patients might look very different and may need to be measured according to different metrics. Nevertheless, the participants were in agreement that it is sensible to start with a focus on solving small and manageable concrete problems. They discussed that translation would be slow and happen first in incremental steps, such as improving prediction of treatment outcomes, or improving current treatments. Furthermore, these concrete problems can provide test cases to work out a common language to bridge the gap between fields.

Indeed, the workshop participants identified the importance of working toward a common language to describe variables and to capture possible disagreements about the underlying computational theories of mental constructs that define how the brains of patients work and how patients behave. Developing a common language is critical to bring together the complicated and diverse components of clinical work, computational approaches, and experimental neuroscience, and to translate the mechanistic understanding that computational approaches can provide (at multiple levels of analysis) into clinical consequences. Although there were contrasting opinions about what that language should be, the workshop did underscore some common terms and concepts that were emerging within component fields, such as attractor networks and state representations, sensory perception and attention, and reinforcement learning.

The workshop participants also identified a need to develop a sustainable infrastructure that establishes standards for data (collection, analytics, housing, sharing, identifying common elements, etc.). Because of the multidisciplinary nature of the field, sharing of raw clinical—as well as other—data in standardized formats is essential for validation and replication. The workshop recommended the construction of benchmark tests and consortia to evaluate the success of translated tools, similar to previous approaches in psychiatry (such as MATRICS [8]).

The primary step that workshop participants recommended for improving translation, however, was cross-disciplinary training to provide computational scientists with clinical training and clinicians with computational training. The development of such transdisciplinary scientists was viewed as a key requirement for the field to be successful. One recommendation was to fund consortia that require interdisciplinary mentorship, as well as “boot camp” meetings to raise interest in the field (see the Computational Psychiatry mini-symposium held at the 2017 Society for Neuroscience (SfN) meeting, and the Explainable Artificial Intelligence meeting). In computational neuroscience, for instance, collaborations between computational scientists and neuroscientists, through summer workshops, boot camps, and internships, eventually led to a new generation of investigators with extensive computational training capable of running experimental laboratories. Creating opportunities for both junior and senior computational scientists to receive clinical experience (e.g., exposure to patients)—without requiring a full training sequence—was deemed critical. Such clinical exposure would ideally enable a better understanding of the clinical language, questions, and data, while also  promoting an appreciation for the limitations and complexity of clinical data. Similarly, opportunities for junior and senior clinicians to receive enough computational training to appreciate and use, if not develop, computational approaches were viewed as key. Eventually, a new generation of clinician scientists will need to be trained. In the meantime, opportunities for current scientists to do month, semester, or year-long internships was suggested as an important step.

## Computation

In addition to the challenges of harmonizing diverse realms (clinical practice/research and quantitative/modeling expertise) into a cohesive field, workshop participants identified several challenges and opportunities within the space of computation itself. It has become clear that computational approaches are a key to understanding how properties inter-relate across levels of analysis (e.g., molecular, neuronal, circuit, system, behavioral, societal), particularly as these levels can interact in complex ways (e.g., societal changes affect behavior, which affects learning, which is stored as molecular changes in neurons and circuits) [4]. There was a general consensus that all levels of analysis are important, as is the integration between them.

Computational psychiatry can also be segregated into theory-driven and data-driven approaches. Theory-driven approaches invoke formal models of brain/behavior relationships to develop and test specific mechanistic hypotheses, which in turn can be examined for their utility in predicting psychopathological outcomes. Data-driven approaches typically utilize large data sets and sophisticated mathematical techniques to characterize either the latent organization of the data (i.e., unsupervised learning), or multivariate relationships between specified groups of variables (i.e., supervised learning). Of course, these approaches are not mutually exclusive; data-driven approaches contain theories, and theory-driven approaches involve data. For instance, what data are collected and how they are collected depends on *a priori* theoretical constructs that guide variable selection. Thus, data collection is never “atheoretical.” In addition, the latent structure found by data-driven machine-learning algorithms can be used to suggest potential theories and theoretical constructs, which must be tested against independent large data sets. The workshop participants agreed that the ultimate aim is to combine these approaches to improve patient outcomes and to further our understanding of psychiatric illnesses.

Workshop participants also suggested that computational psychiatry may be able to reconcile psychiatric illness with specific neuroscience constructs such as those arising from decision-making sciences [9–12] or from efforts to characterize psychopathology in terms of multiple dimensions through the NIMH Research Domain Criteria (RDoC) [13, 14]. RDoC is an integrative framework with the goal of mechanistically explaining domains of mental function, behaviors, and symptoms that can then be used to identify “failure modes,” or potential causes of dysfunction, within those domains. In the same way that RDoC provides a framework to connect psychological constructs with neuropsychiatric domains, there are a number of similar conceptual frameworks that have been proposed. For instance, in the decision-making literature, reinforcement learning provides a framework to explain behavioral actions and their failure modes. The reinforcement learning literature has also begun to incorporate more complex phenomena and other psychological constructs beyond simply action-reward relationships (e.g., situation-recognition, event categorization, and social and moral interactions). How to translate between RDoC and these other computational frameworks, and whether those frameworks can explain the heterogeneity in human psychiatry, remains an open question.

## Psychiatry

A key conceptual framework in psychiatry that might benefit from computational approaches is its nosology, which is its diagnostic or classification system. The most commonly used nosology in psychiatry is the Diagnostic and Statistical Manual of Mental Disorders (DSM) framework, a system for making reliable diagnoses using signs and symptoms organized as summaries of clinical observations that often occur together. As described in the DSM, psychiatric disorders are composed of multiple symptoms that can be measured independently, along multiple dimensions. RDoC, although  not a nosology, is a conceptual framework informed by an understanding of neural systems that can accommodate such multidimensionality and can be linked to DSM observations through the concept of the “failure mode,” in which one identifies potential causes leading to particular dysfunctions in a system, in this case, a neural system.

An important complexity in the nosology of psychiatry is that brain dysfunctions seem to lead to psychiatric dysfunctions with both multifinality (the same cause leads to multiple outcomes) and multipotentiality (the same outcome arises from multiple causes). Computational methods can be used to reason about these complex relationships. For example, the recent Strungmann Forum on Computational Psychiatry [3] proposed using Bayesian inference to link underlying causes (genetics, brain circuits, and sociological phenomena), latent hypothesized theoretical constructs, and symptoms. Bayesian inference allows reasoning through many-to-many mappings both from causes to constructs to symptoms and from symptoms to constructs to causes. Certainly, RDoC’s multiple levels of analysis can also provide a platform for this type of reasoning and computational approaches can provide mathematical tools to link these different levels of analysis. Workshop participants agreed that these links were a key step toward the target of precision medicine because a more biologically based nosology is likely to provide more precise mechanistic interventions and treatments.

A major challenge in contemplating these endeavors is the heterogeneity of psychiatric data sets, which stems from several factors. One is the inherent noisiness of currently defined psychiatric diagnoses, which are constructed from observations about symptom patterns rather than underlying structural and functional neuropathology. Moreover, individual differences in biological systems and sub-threshold psychopathology are apparent even in psychiatrically healthy subjects. Finally, psychiatric dysfunction usually arises from interactions among multiple causes, including genetic, environmental, and social, all translated through neural and developmental processes. However, there are also sources of heterogeneity that arise from attributes of the data itself. The methods for data collection (e.g., genetic versus imaging), the frequency of the measurements and degree of missingness (in terms of, for instance, sparse or missing data) all contribute to the heterogeneity and produce challenges for merging diverse data sets. Novel strategies for addressing this heterogeneity, reducing dimensionality, and analyzing data will be key as computational psychiatry grows as a field. Computational neuroscience provides analytic tools that can help address these challenges.

Furthermore, a significant proportion of current data collection is based on subjective reports collected through questionnaires. Workshop participants agreed that subjective experience is inherent to psychiatric phenomena. Nevertheless, there was consensus that it was also critically important to examine factors related to psychopathology that could be measured objectively, through purely behavioral or neurophysiological inputs. New ecologically valid measures that allow participants to generate data outside of a laboratory setting, including behavioral signs such as social media activity, are now becoming available. In addition, studies can leverage ecological momentary assessments and physiological measures such as skin conductance or cortisol response in stress paradigms, that may be related closely to neural systems, but are not currently used as standard clinical tools. Participants agreed that the array of assessments should be expanded to include both behaviors and neurophysiological assessments that can capture symptom severity, subjective states, etc., while also capturing contexts (social, functional, stress, medical, etc.). It will be important to ensure that these assessments be developed with computational techniques in mind to mitigate their inherent heterogeneity. To ensure that computational psychiatry research uses the best and most relevant computational strategies to scientifically address a clinical question, engineers and data scientists need to be involved from the earliest planning phases. Both data-driven and theory-driven approaches require samples that are representative of the population of interest and sized so as to allow for adequate power.

## Basic/fundamental science

Workshop participants noted that understanding the pathological mechanisms that give rise to psychiatric disorders requires work in model systems, where invasive techniques can be used and theoretically driven experiments can be done. One concept discussed in the workshop was the usefulness of both causal translation and functional translation. Causal translation means that hypothesized causes of a disorder are replicated in a non-human model system, for example, changing the genetics of a mouse to explore the neural and behavioral consequences of mutations known to exist in subjects with autism, or removing a pup from its mother’s care as a model of maternal neglect. Functional translation is when the effect of the disorder is constructed in a non-human model system, for example, lesioning the dopamine system as a model of Parkinson’s disease or using long-term potentiation to modulate a neural circuit that may be dysfunctional in schizophrenia. Several workshop participants raised the issue that success at improving treatments using studies in model systems has been limited [5, 15], but other workshop participants suggested that taking a longer view can provide examples of successes in these realms. Computation can also bridge the gap between mechanisms and related metrics studied in humans and non-human animals. The workshop participants noted that it was important to have bidirectional information flow not only from the bench to the bedside, but also from the bedside to the bench. The cross-disciplinary training and development of a common language noted at the beginning of this document is a particularly important first step to enabling this bidirectional translational pipeline.

To make progress through model systems, it is necessary to establish homology between neural systems in human and non-human animals. Of note, if an animal solves a cognitive task using a different behavioral strategy and neural system—if it has evolved a different strategy because of its ecological niche—it may not be possible to translate from studies in the model systems to patients. It is, therefore, important to carefully translate neural mechanisms and not just behaviors between human and non-human animals. Intuition and semantic similarity about whether a behavior in a model system engages the same neural system as a similar behavior or construct in human subjects can be misleading. Rigorous vetting of the homology of behaviors and the involved neural systems is essential. Nevertheless, such homologous neural systems driving behaviors do exist and there are numerous cases of successful homological translation in sensory, motor, and cognitive domains.

Related to the problem of developing behaviors and their associated computational formalisms that span species is the problem of closing the gap between questionnaires and other clinical tools that are typically used in psychiatry, and the behavioral and computational approaches that are used in non-human animal models. Questionnaires generate reliable information on the presence or absence of common clinical features of psychiatric disorders. These instruments do not translate easily to the systems neuroscience approaches typically used in non-human animal models. Further, questionnaires do not always correlate with task-based methods even when they putatively measure similar constructs. Crossing this gap will require the development of a set of experimental measurements, for example behavioral tasks and non-invasive neuroimaging, that provide the same or better diagnostic utility as clinical tools. The advantage of such a set of metrics is that they would correspondingly provide insight into the neural mechanisms that give rise to the disorders. However, understanding of mechanism will not necessarily lead to better treatments quickly. Current treatments, including pharmaceutic and brain stimulation approaches, only provide coarse manipulations of what are subtle, sophisticated, and complex systems. Development of approaches that allow for more sophisticated and subtle manipulations of neural systems are underway, such as methods for targeted delivery of pharmacologic, behavioral, and neuromodulatory interventions. A rational approach to these new interventions will require an understanding of not only how neural systems work but also how those new interventions change the function of neural systems.

The use of RDoC and other conceptual frameworks as the means of framing experimental questions in human subjects may allow for better comparison between animal and human data because the data from both subjects would be taken at the level of translatable constructs (working memory, attention, etc.) rather than trying to find animal models of DSM diagnoses. An understanding of the behaviors mediated by circuitry relevant to psychiatric disorders may further improve dimensional approaches. New tools to interrogate circuits in both human and non-human animals are becoming available almost daily.

## Conclusion

This workshop identified several challenges and opportunities for the nascent field of computational psychiatry, most importantly requiring the bolstering and deepening of existing collaborative approaches and opportunities, and the creation of new ones. Creation of a common language will require at least some common understanding, with clinical/translational researchers taking the step across the computational barrier and vice versa, but will be key to successful collaborations. Funding opportunities, training programs (formal or informal), and further scientific meetings that bring together these diverse and important perspectives will be critical. Work will need to be done refining the conceptual frameworks and determining the relationship between the conceptual frameworks (such as RDoC) and psychiatric dysfunction (such as through newly-identified failure modes or through classical classifications such as DSM). Theory- and data-driven approaches will need to be integrated, likely in a cyclical manner of refinement, through the creation of a novel common language spoken by the new trainees in computational psychiatry.
